# The Unfolded Protein Response and the Role of Protein Disulfide Isomerase in Neurodegeneration

**DOI:** 10.3389/fcell.2015.00080

**Published:** 2016-01-08

**Authors:** Emma R. Perri, Colleen J. Thomas, Sonam Parakh, Damian M. Spencer, Julie D. Atkin

**Affiliations:** ^1^Department of Biochemistry and Genetics, La Trobe Institute for Molecular Science, La Trobe UniversityMelbourne, VIC, Australia; ^2^Department of Physiology, Anatomy and Microbiology, School of Life Sciences, La Trobe UniversityMelbourne, VIC, Australia; ^3^Department of Biomedical Sciences, Faculty of Medicine and Human Science, Macquarie UniversitySydney, NSW, Australia

**Keywords:** endoplasmic reticulum stress (ER stress), unfolded protein response (UPR), protein disulfide isomerase (PDI), neurodegeneration, Alzheimer's disease (AD), Parkinson's disease (PD), amyotrophic lateral sclerosis (ALS), Huntington's disease (HD)

## Abstract

The maintenance and regulation of proteostasis is a critical function for post-mitotic neurons and its dysregulation is increasingly implicated in neurodegenerative diseases. Despite having different clinical manifestations, these disorders share similar pathology; an accumulation of misfolded proteins in neurons and subsequent disruption to cellular proteostasis. The endoplasmic reticulum (ER) is an important component of proteostasis, and when the accumulation of misfolded proteins occurs within the ER, this disturbs ER homeostasis, giving rise to ER stress. This triggers the unfolded protein response (UPR), distinct signaling pathways that whilst initially protective, are pro-apoptotic if ER stress is prolonged. ER stress is increasingly implicated in neurodegenerative diseases, and emerging evidence highlights the complexity of the UPR in these disorders, with both protective and detrimental components being described. Protein Disulfide Isomerase (PDI) is an ER chaperone induced during ER stress that is responsible for the formation of disulfide bonds in proteins. Whilst initially considered to be protective, recent studies have revealed unconventional roles for PDI in neurodegenerative diseases, distinct from its normal function in the UPR and the ER, although these mechanisms remain poorly defined. However, specific aspects of PDI function may offer the potential to be exploited therapeutically in the future. This review will focus on the evidence linking ER stress and the UPR to neurodegenerative diseases, with particular emphasis on the emerging functions ascribed to PDI in these conditions.

## Introduction

The endoplasmic reticulum (ER) is a fundamental cellular organelle responsible for the folding, post-translational modification, transportation, and quality control of newly synthesized proteins. The ER is therefore a key component of cellular protein homeostasis, or proteostasis, integrated mechanisms that control the regulation of protein trafficking, synthesis, folding, and degradation. The maintenance and regulation of proteostasis is a critical function for post-mitotic cells such as neurons and dysregulation of proteostasis is increasingly implicated in diseases that target neurons, including neurodegenerative diseases. A pathological hallmark of these diseases is the accumulation of misfolded protein aggregates within affected neurons. Whilst neurodegenerative diseases differ in the proteins which misfold, and the sub-groups of neurons affected, abnormal protein misfolding is a common feature.

When the accumulation of misfolded or unfolded proteins occurs within the ER, this disturbs ER homeostasis, giving rise to ER stress. ER stress results in activation of the unfolded protein response (UPR) which aims to alleviate the stress. The UPR involves up-regulation of protein chaperones to promote protein folding, translational attenuation to reduce the load of proteins within the ER to prevent further accumulation of misfolded proteins, and up-regulation of ER-associated protein degradation (ERAD) and autophagy to promote degradation of misfolded proteins. Therefore, ER stress plays a pivotal role in cell survival by maintaining proteostasis. In circumstances of chronic or prolonged ER stress, however, the UPR becomes pro-apoptotic, therefore triggering cell death. ER stress is increasingly implicated as a key mechanism relevant to pathogenesis in neurodegenerative diseases, although differential effects are evident in different neurodegenerative conditions.

Chaperones promote the correct folding of proteins into their native conformations and hence are an important mechanism in proteostasis. One chaperone upregulated during the UPR is Protein Disulfide Isomerase (PDI), which is found primarily within the ER, but is also found in other cellular locations. PDI is the prototype of a family of proteins that possess two alternative functions; general chaperone activity and disulfide interchange activity; in which protein disulfide bonds are oxidized, reduced, or isomerized. As chaperones prevent protein misfolding, novel therapeutic strategies mimicking the functional activity of PDI may therefore be beneficial in disorders involving protein misfolding. Consistent with this notion, there is increasing evidence linking PDI to neurodegenerative diseases. Recent studies have revealed unconventional roles for PDI in these disorders, distinct from its normal function in the UPR and the ER, although these mechanisms remain poorly defined. This review will focus on the evidence linking ER stress and the UPR to neurodegenerative diseases, with particular emphasis on the role of PDI in these conditions.

## Neurodegeneration

**Figure 1:**
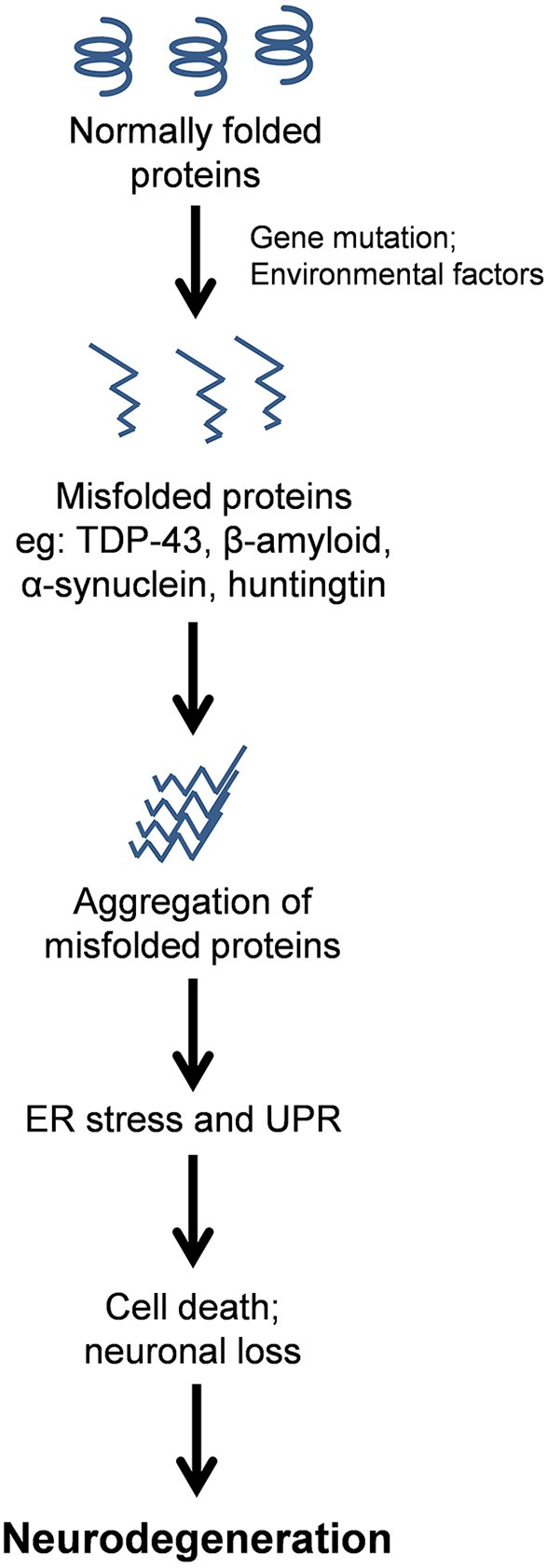
**Schematic representation of the protein pathology contribution to neurodegenerative disease via chronic Endoplasmic Reticulum (ER) stress**. Misfolded proteins aggregate and form prominent inclusions as the characteristic pathological hallmark of these disorders. ER stress results from the accumulation of misfolded proteins within the ER, thus activating the Unfolded Protein Response (UPR), which if prolonged or unresolved, can result in neuronal cell death, and hence neurodegenerative disorders.

Neurodegenerative diseases have long been regarded as intangible mysteries of biomedical research and targeting these conditions therapeutically remains a major obstacle in medicine. Whilst these disorders are distinct in their clinical manifestations, they share a common pathological hallmark: the abnormal aggregation of misfolded proteins (Lindholm et al., 2006). Aggregation occurs when misfolded proteins expose hydrophobic regions that are normally hidden within the protein interior when folded in their native conformation. This exposure of normally buried regions promotes hydrophobic interactions with other proteins. Protein misfolding is triggered by genetic mutations in familial forms of disease, or by cellular conditions which cause wildtype proteins to misfold in sporadic forms of disease, although the latter processes remain poorly defined. The aggregation of misfolded proteins leads to the formation of prominent protein inclusions (Wolozin, 2012; Figure 1). The most prevalent neurodegenerative diseases include Alzheimer's disease (AD), Parkinson's disease (PD), amyotrophic lateral sclerosis (ALS), Huntington's disease (HD), and transmissible prion encephalopathies, such as Creutzfeldt-Jakob disease (CJD). These disorders differ in the proteins that misfold and the group of neurons which are affected (Soto, 2003; Table 1). An intriguing puzzle is why specific groups of neurons are selectively targeted in these conditions when the proteins that misfold are usually expressed ubiquitously in all cell types. At the biochemical level, however, these disorders share a common mechanism; formation of abnormal misfolded protein aggregates which lead to the protein inclusions characteristic of pathology in these disorders.

**Table 1:** **Genes and proteins implicated in common neurodegenerative diseases**.

**Neurodegenerative disease**	**Genes implicated in disease**	**Proteins encoded**	**References**
Alzheimer's disease	APP	Amyloid precursor protein	Haass and Selkoe, 2007; Karran et al., 2011; Matus et al., 2011
	PSEN1	Presenilin-1	
	PSEN2	Presenilin-2	
	MAPT	Tau	
Parkinson's disease	SNCA PARK2 PINK1 PARK7 UCH-L1 PARK8	α-Synuclein Parkin PTEN-induced putative kinase 1 DJ-1 Ubiquitin carboxyl-terminal esterase L1 LRRK2	Chartier-Harlin et al., 2004; Schlehe et al., 2008; Volpicelli-Daley et al., 2011
Amyotrophic lateral sclerosis	SOD1	Cu/Zn superoxide dismutase 1	Ferraiuolo et al., 2011; Turner et al., 2013; Leblond et al., 2014; Renton et al., 2014
	TARDBP	TAR DNA binding protein 43 (TDP-43)	
	FUS	Fused in sarcoma	
	C9orf72	Chromosome 9 open reading frame 72	
	ALS2	Alsin	
	SETX	Senataxin	
	VAPB	Vesicle-associated membrane protein-associated B	
	OPTN	Optineurin	
	VCP	Valosin-containing protein	
	UBQLN2	Ubiquilin 2	
	PFN1	Profilin 1	
	SQSTM1	Sequestosome 1	
	HnRNPA2B1/A1	Heterogenous nuclear ribonucleoprotein	
	TAF15	TATA box binding protein-associated factor	
Huntington's disease	HTT	Huntingtin	Carnemolla et al., 2009; Ross and Tabrizi, 2011
Creutzfeldt-Jakob disease	PRNP	PrP protein	Head and Ironside, 2012; Porter and Leemans, 2013

### Alzheimer's disease

AD is characterized by a progressive decline in memory, language, behavior, and cognitive function (Salminen et al., 2009). Neuronal degeneration in AD occurs in the cerebral cortex (in particular the frontal, temporal, and parietal lobes) and the hippocampus (Brundin et al., 2010; Fjell et al., 2014). Two types of misfolded protein inclusions are present in these tissues. Amyloid plaques are formed extracellularly by the aggregation of β-amyloid, which is produced by abnormal cleavage of amyloid precursor protein (APP), and neurofibrillary tangles, which are formed intracellularly in the cytoplasm by hyper-phosphorylation of the microtubule associated protein tau (Haass and Selkoe, 2007; Matus et al., 2011). Mutations that cause familial cases of AD occur in genes encoding APP, or the presenilin genes (PS1 and PS2) which encode proteins that comprise the secretase complex that regulates APP processing. These mutations alter the metabolism of β-amyloid, which either increase the total production of β-amyloid or reduce its rate of degradation. The increased levels of β-amyloid then lead to its oligomerization (Haass and Selkoe, 2007; Karran et al., 2011).

### Parkinson's disease

PD results from the degeneration of dopaminergic neurons primarily in the *substantia nigra pars compacta* in the midbrain (Matus et al., 2011). As a consequence, PD patients experience symptoms of motor dysfunction; tremors, bradykinesia (slowed movement), rigidity, loss of autonomic movement, and abnormal gait (Jankovic, 2008). The most widely studied gene linked to PD encodes α-synuclein, which causes rare autosomal dominant familial forms of disease (Chartier-Harlin et al., 2004). α-synuclein is found in both Lewy bodies and Lewy neurites, which are protein inclusion bodies found in the neuronal cytoplasm and processes, respectively (Volpicelli-Daley et al., 2011). Mutations in the genes encoding Parkin, PINK1, and DJ-1, LRRK2 and UCH-L1 cause familial forms of PD, which arise through autosomal recessive inheritance (Schlehe et al., 2008).

### Amyotrophic lateral sclerosis

ALS is characterized by the degeneration of upper motor neurons of the motor cortex and lower motor neurons of the brainstem and spinal cord (Kent-Braun et al., 1998). Patients typically experience symptoms of fatigue, muscle weakness and atrophy, followed by paralysis (Rothstein, 2009; Robberecht and Philips, 2013). There are numerous genes linked to ALS both genetically and/or pathologically (Leblond et al., 2014; Renton et al., 2014; Table 1). Protein inclusions in ALS form in the cytoplasm of degenerating motor neurons and depending on the patient, contain primarily [Cu/Zn] superoxide dismutase 1 (SOD1), TAR DNA binding protein 43 (TDP-43), fused in sarcoma (FUS), or by dipeptide repeat proteins produced by non-conventional repeat associated non-ATG translation, encoded by the Chromosome 9 open reading frame 72 repeat expansion (C9orf72) (Ferraiuolo et al., 2011; Turner et al., 2013; Leblond et al., 2014; Renton et al., 2014). Recent evidence has demonstrated that ALS is linked to closely to frontotemporal dementia (FTD) (Ling et al., 2013).

### Huntington's disease

Characterized by involuntary movements such as twitching and chorea (jerky movements of limbs), personality changes and dementia, HD is an autosomal dominant disease (Brundin et al., 2010). Mutations in the huntingtin (Htt) gene result from the expansion of a CAG repeat, which leads to an aberrantly long polyglutamine sequence in the huntingtin protein (Ross and Tabrizi, 2011). Huntingtin proteins with less than 35 polyglutamine repeats do not aggregate readily, however proteins with more than 40 repeats result in aggregation into inclusion bodies (Brundin et al., 2010). In general, longer lengths of polyglutamine repeats result in more rapid neurodegeneration and earlier disease onset than shorter repeats (Ross and Tabrizi, 2011). In contrast to the other neurodegenerative diseases in which most cases (85–90%) are sporadic, HD is entirely genetic in nature (Carnemolla et al., 2009).

### Creutzfeldt-Jakob disease and other prion encephalopathies

Transmissible prion encephalopathies are neurodegenerative disorders in which proteins become infectious and protein misfolding propagates from one cell to another. These infectious proteins are termed prions (Makarava et al., 2012). The most common transmissible prion encephalopathy is CJD, in which patients experience memory loss, cognitive decline, personality changes, and psychosis (Porter and Leemans, 2013). The prion gene PRNP encodes cellular prion protein (PrP^c^), which occurs naturally in both humans and animals. However, in CJD, the accumulation of abnormal prion protein, the scrapie isoform PrP^Sc^, results, which is the major component of the purified infectious agent (Head and Ironside, 2012). PrP^Sc^ promotes refolding of natively folded PrP^c^ proteins into disease-associated misfolded PrP^Sc^ prions, resulting in insoluble protein inclusions in the brain and lympho-reticular tissues (Porter and Leemans, 2013). Not surprisingly, familial forms of CJD are caused by mutations in the PRNP gene (Head and Ironside, 2012).

Proteins misfold as part of normal cellular physiology, however normally, cells do not accumulate protein aggregates. Hence, what causes the formation of protein inclusions in neurodegeneration? A striking feature of neurodegenerative diseases is that they are late onset, and the probability of disease onset rises significantly with age. Therefore, pathology can be hypothesized to arise as a consequence of the normal aging process, whereby proteostasis becomes increasingly more difficult for cells to maintain as misfolded proteins continuously accumulate within the neuron. These mechanisms are not well understood, although decreases in chaperone activity or the efficiency of protein degradation processes over time may accelerate the accumulation of misfolded proteins (Nakamura and Lipton, 2011). Similarly, a decrease in antioxidant defenses during normal aging results in increases in the production of free radicals, therefore promoting oxidative stress (Halloran et al., 2013). Oxidative stress ultimately damages cells and is closely associated with ER stress (Lindholm et al., 2006; Kanekura et al., 2009). We describe below the increasingly complex relationship between the ER and protein misfolding in the context of age-related neurodegenerative disorders.

## ER stress

### The Endoplasmic Reticulum (ER) in neurons

The ER is the largest membrane-bound organelle in the cell, and it possesses a diverse range of signaling and homeostatic functions. As well as the synthesis, folding/maturation, and trafficking of all secretory/transmembrane proteins, it also synthesizes lipids, plays a critical role in Ca^2+^ homeostasis, and is essential for compartmentalization of the nucleus and the structure of chromatin. Recently, novel ER functions have been described, including the regulation of mitochondrial function and the formation of autophagosomes, thus highlighting the importance of this organelle in cellular organization and proteostasis. The ER is well characterized in yeast and specialized secretory cells, but the ER in neurons is poorly studied in comparison. It is clear, however that the ER is much more extensive in neurons than in other cells, extending throughout the entire dendritic arbor and axon (Ramirez et al., 2011). There is evidence that multiple proteins are synthesized locally in both dendrites and axons (Lin and Holt, 2008; Yudin et al., 2008; Merianda et al., 2009). However, in these compartments, in particular within the axon, the ER remains largely uncharacterized.

Equipped with a variety of chaperones and folding enzymes, the ER maintains tight quality control measures to sustain its environment (Halperin et al., 2014). When nascent proteins enter the ER, the formation of disulfide bonds and other post-translational modifications facilitate correct protein folding. Disulfide bond formation is catalyzed by the PDI family of proteins and is possible due to the oxidizing environment of the ER (E°′ = −0.18*V*; Woycechowsky and Raines, 2000; Wilkinson and Gilbert, 2004). These disulfide bonds ensure protein structural stability and promote assembly of multi-protein complexes (Woycechowsky and Raines, 2000). Correctly folded proteins are transported to the Golgi apparatus, where they are further sorted, modified and then packaged for secretion. The ER-Golgi route is an important cellular pathway; one third of all proteins transit the ER and Golgi compartments destined for transmembrane, ER or extracellular locations (Ghaemmaghami et al., 2003). Proteins which are unable to be correctly folded are directed to Endoplasmic Reticulum-Associated Degradation (ERAD), where misfolded proteins are transported to the cytosol for degradation by the proteasome, or by autophagy (Hetz and Mollereau, 2014).

### The unfolded protein response

**Figure 2:**
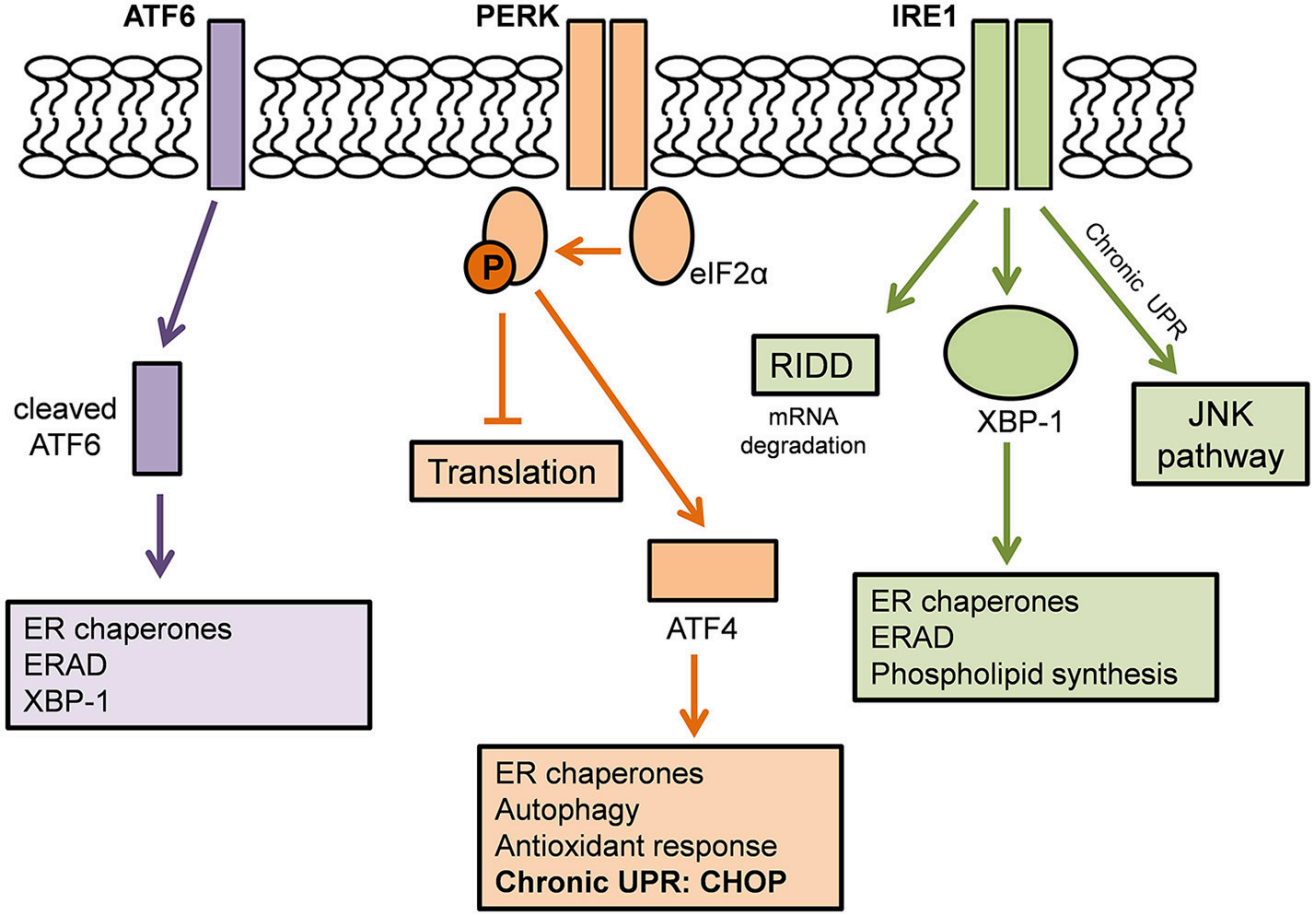
**The Unfolded Protein Response**. Adapted from Hetz and Mollereau (2014). A schematic demonstrating the action of the three ER stress sensors on the Unfolded Protein Response.

The UPR is an adaptive mechanism designed to cope with protein folding alterations in the ER, and thus restore proteostasis (Walker, 2010). Under moderate misfolded protein accumulation, the UPR transduces information from the ER to the nucleus and cytosol and thereby inhibits protein translation, expands the ER membrane, recruits ER chaperones to aid in the correct folding of misfolded proteins, and promotes protein degradation in order to reduce the load of unfolded or misfolded proteins (Hetz, 2012). However, under conditions of chronic or irreversible ER stress, such as in disease states, the UPR shifts from being protective to pro-apoptotic, and multiple integrated apoptotic pathways can trigger cell death (Figure 2).

The UPR is mediated by three ER stress sensors; PKR-like endoplasmic reticulum kinase (PERK), inositol-requiring kinase 1 (IRE1), and activating transcription factor 6 (ATF6). These ER stress sensors are bound to the ER chaperone, BiP, under basal conditions, keeping them in an inactivated state. When ER stress arises, for example, there is an accumulation of misfolded proteins in the ER lumen, BiP dissociates from the ER stress sensors to preferentially bind the hydrophobic regions of the misfolded proteins, thus resulting in their activation (Rutkowski et al., 2006).

In one pathway, upon activation, PERK directly phosphorylates, and thus inhibits, the ubiquitous eukaryotic translation initiation factor 2α (eIF2α). As a consequence, there is a reduction in the entrance of newly synthesized proteins into the ER lumen, therefore reducing the load of ER protein-folding (Boyce et al., 2005). Phosphorylation of eIF2α also favors the selective translation of the mRNA encoding the transcription factor ATF4. ATF4 translocates to the nucleus where it induces the expression of ER chaperones, such as PDI, which increases refolding of misfolded proteins (Halperin et al., 2014). ATF4 also induces the expression of various genes involved in autophagy, antioxidant response, and amino acid biosynthesis and transport (Cao and Kaufman, 2012; Hetz and Mollereau, 2014).

A second pathway of the UPR is initiated by IRE1 which is activated upon its dimerization and auto-phosphorylation (Hetz, 2012). IRE1 degrades a subset of mRNAs which encode for ER-localized proteins by means of regulated IRE1 dependent decay (RIDD), thereby reducing protein synthesis in the ER (Cao and Kaufman, 2012). IRE1 also catalyses the splicing of the mRNA encoding transcription factor X-box binding protein 1 (XBP-1). This splicing removes a 26 base intron from XBP-1, resulting in a shift in the reading frame of its mRNA (Hetz and Mollereau, 2014). Spliced XBP-1 is a stable transcription factor which translocates to the nucleus to induce the upregulation of ER chaperones and proteins involved in the ER-associated degradation (ERAD) pathway. XBP-1 also controls phospholipid synthesis which is important for ER membrane expansion when the ER is under stress (Lee et al., 2003).

In a third pathway, upon UPR activation, ATF6 translocates from the ER membrane to the Golgi apparatus where it is cleaved by site-1 and site-2 proteases. The resulting cytosolic ATF6 fragment (ATF6f) translocates to the nucleus to induce gene expression of ER chaperones, ERAD components and XBP-1 (Schröder and Kaufman, 2005). Together, the ER stress sensors regulate the expression of a number of overlapping target genes, encoding for proteins which modulate adaptation to stress, thus promoting the survival of the cell.

### Chronic activation of the UPR

Under conditions of chronic or irreversible ER stress, such as those that occur in disease, there is a shift in the paradigm of the UPR from being pro-protective to pro-apoptotic. The UPR therefore induces apoptosis mediated by overlapping apoptotic signaling mechanisms (Ma and Hendershot, 2004). Sustained activation of the ER stress sensor PERK elicits a chain of transcriptional responses mediated by ATF4. ATF4 induces the upregulation of the transcription factor C/EBP-homologous protein (CHOP) and its target growth arrest and DNA damage-inducible 34 (GADD34). The promoter of CHOP contains the binding sites for several players of the UPR, including ATF4 and ATF6, and is a key mediator of ER stress induced apoptosis (Cao and Kaufman, 2012). CHOP can inhibit the expression of survival protein BCL-2 and simultaneously engage pro-apoptotic proteins such as Bcl2-interacting mediator of cell death (BIM) and p53 upregulated modulator of apoptosis (PUMA). The outcome is the activation of BAX- and BAK- dependent apoptosis at the mitochondria and the caspase cascade, which result in apoptosis of the cell (Soo et al., 2009; Hetz and Mollereau, 2014). Furthermore, CHOP induces ERO1α which causes oxidative stress by transferring electrons from PDI to O_2_ to produce hydrogen peroxide. The activation of GADD34 results in the dephosphorylation of eIF2α, thus increasing protein synthesis and accentuating the ER and oxidative stress (Cao and Kaufman, 2012). IRE1 also plays a role in apoptosis of a cell through the recruitment of apoptosis signaling kinase (ASK1) which activates the c-Jun N-terminal kinase (JNK) pathway, which stimulates proinflammatory responses apoptotic pathways (Nishitoh et al., 2002), and also by direct interaction with BAX and BAK (Hetz et al., 2006). It is this apoptosis of cells which results in the degeneration of motor neurons characteristic of neurodegenerative diseases.

The UPR therefore constitutes a complex mechanism of integrated signaling pathways that responds to ER stress by either cellular adaptation, thus promoting the survival of the cell, or by triggering apoptosis. Similar to other aspects of neuronal ER biology, whilst the UPR is well-characterized in many cell types, its function in neurons is poorly understood (Wang and Kaufman, 2012) and the response of the UPR in the axonal and dendritic compartments remains uncharacterized. Similarly, it is unknown if the expression of UPR proteins is induced locally or somally in response to neuronal activity. Whilst mechanisms underlying the selective vulnerability of neurons to degeneration remain unknown, an interesting possibility is that the unique properties of the ER may contribute and render neurons particularly sensitive to ER stress. However, this possibility remains largely unexplored.

### Protein Disulfide Isomerase

#### Structure and expression of PDI

**Figure 3:**
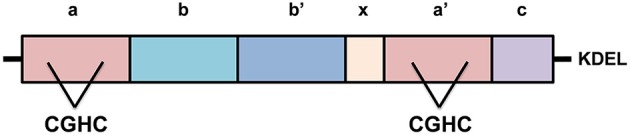
**The structure of Protein Disulfide Isomerase (PDI)**. PDI has four distinct domains, a, a', b, and b', as well as a linker region, “x”, and its C-terminal extension with KDEL sequence. The catalytic domains, a and a', possess active sites containing the motif cysteine-glycine-histidine-cysteine (CGHC). KDEL, the ER retention signal sequence.

PDI is a 55kDa ER chaperone primarily localized in the ER (Ellgaard and Ruddock, 2005), however it has also been detected in the cytoplasm, nucleus and extracellularly in a biologically active state (Turano et al., 2002). PDI has four distinct domains within its structure; a, a', b, and b', with a highly acidic C-terminal extension and an x linker region (Figure 3). An ER-retention signal sequence (KDEL) lies at the C terminus (Hatahet and Ruddock, 2007). The a and a' domains are catalytic domains, similar to thioredoxin, which are separated by the two non-catalytic domains (b and b') (Hatahet and Ruddock, 2007), that only share 16.5% sequence identity (Xu et al., 2014). The catalytic domains contain an active site motif comprising two cysteine residues separated by glycine and histidine (Cys-Gly-His-Cys). The oxidoreductase activity of PDI relies on the thiol groups of these active site cysteines (Jessop et al., 2009). Each CGHC active site has a high disulfide reduction potential (E°′ = −0.18 V) and a low pK_a_ value (pK_a_ = 6.7) making it a competent oxidizing agent in the ER (Woycechowsky and Raines, 2000; Liu et al., 2013; Figure 3).

#### Functions of PDI

**Figure 4:**
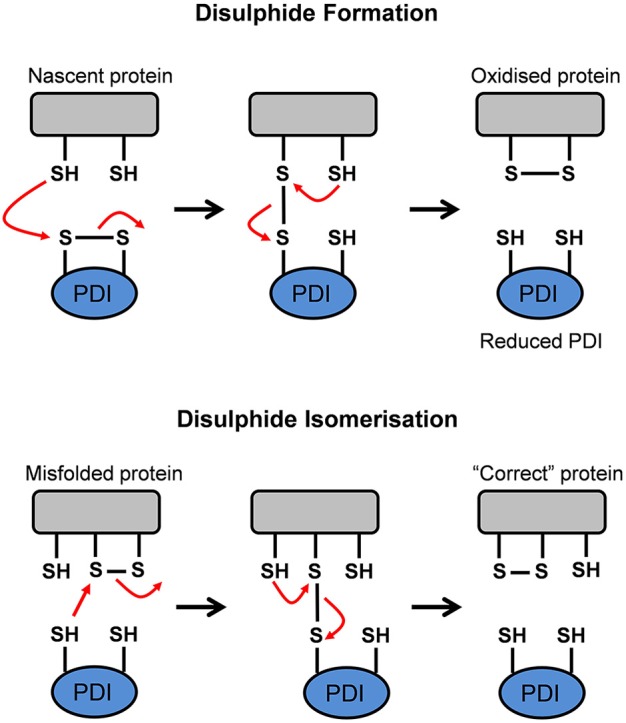
**Schematic representation of the disulfide interchange activity of Protein Disulfide Isomerase (PDI)**. PDI can form, break down, and rearrange disulfide bonds. This aids in promoting the formation of the native conformation in a misfolded or unfolded protein. Adapted from Forrester et al. (2006).

PDI has two major functions. Firstly, it is responsible for the oxidation (formation), reduction (break down) and isomerization (rearrangement) of protein disulfide bonds via disulfide interchange activity (illustrated in Figure 4). Secondly, PDI has general chaperone activity (Ferrari and Söling, 1999).

##### Disulfide interchange activity

The **a** and **a'** domains of PDI operate independently of one another, as disruption to one domain abolishes 50% of catalytic activity. However, disruption to the active site of both domains results in complete elimination of oxidoreductase activity (Xu et al., 2014). The redox state of the active site cysteine residues and the properties of its substrate determine whether PDI acts as an oxidase, reductase, or isomerase (Xu et al., 2014). In an oxidation reaction, whereby PDI mediates oxidative protein folding to form disulfide bonds in a nascent protein, the substrate dithiol is oxidized to a disulfide. The substrate's reduced cysteine thiols bind with the CGHC motif disulfide on PDI to form a PDI-protein complex. A second reduced thiol from the protein substrate interacts with the complex to produce an oxidized protein which is correctly folded and stabilized. Simultaneously, the active site disulfide in PDI is then reduced to the dithiol state (Forrester et al., 2006). When PDI is reduced after donating a disulfide bond to nascent proteins, it is subsequently reoxidized in the oxidizing environment of the ER. Alternatively, cellular regulators, such as endoplasmic reticulum oxidoreductin 1 (Ero1) interacts with, and reoxidises, the reduced PDI (Mezghrani et al., 2001; Medraño-Fernandez et al., 2014). Oxidative protein folding, however, is prone to error and incorrectly folded proteins can arise as part of normal physiology. Hence, non-native disulfide bonds need to be corrected via isomerization, or reduced to produce the native conformation (Wilkinson and Gilbert, 2004). In a reduction reaction, whereby PDI breaks down disulfide bonds in protein substrates, a substrate disulfide is reduced to the dithiol state, while an active site disulfide is formed in PDI. Reductants such as glutathione (GSH) and NADPH donate electrons to reduce the disulfide in PDI back to its dithiol state (Xu et al., 2014). When misfolded proteins form, isomerization of disulfide bonds is required to convert the disulfides to their native conformation. To facilitate isomerization, one of PDI's active sites must be in a reduced state (Medraño-Fernandez et al., 2014). Isomerization, or the rearrangement of disulfide bonds in a substrate protein, is initiated by the cysteine nearest the N-terminus at each PDI active site (CGHC). This cysteine binds a substrate disulfide which results in an intramolecular rearrangement within the substrate itself. Conversely, isomerization can be seen as repeated cycles of reduction and oxidation (Wilkinson and Gilbert, 2004). Ultimately, the impairment of PDI's activity can lead to the accumulation of misfolded proteins, resulting in ER stress and activation of the UPR (Forrester et al., 2006).

##### Chaperone function

Chaperone binding keeps proteins soluble and competent to fold in order to acquire their native structure (Kojer and Riemer, 2014). As a chaperone, PDI binds to misfolded proteins to prevent them from aggregating and targets misfolded proteins for degradation (Ma and Hendershot, 2004). Although all of the domains of PDI contribute to the binding of misfolded proteins, the **b'** domain comprises the principal substrate-binding site, utilizing hydrophobic interactions to exhibit high affinity and broad specificity (Xu et al., 2014).

#### S-nitrosylation of PDI

Cellular redox states normally regulate cellular function and maintain homeostasis, but when redox homeostasis is disturbed, neurodegeneration can result. The ER is able to withstand mild insults of stress, however a build-up of reactive oxidative species (ROS) and reactive nitrogen species can result in oxidative and nitrosative stress in the ER (Halloran et al., 2013). Neurons are particularly vulnerable to redox dysregulation due to their large size and high oxygen consumption (Parakh et al., 2013). In addition, normal antioxidant defenses usually decline during the normal aging process and hence nitrosative and oxidative stress increases, thus rendering neurons susceptible to age-related degenerative conditions (Halloran et al., 2013). The excessive generation of nitric oxide (NO) has been implicated in AD, PD, and ALS (Forrester et al., 2006). In AD, elevated levels of oxidative and nitrosative stress are associated with alterations in amyloid-β metabolism (Mangialasche et al., 2009).

In conditions of elevated nitrosative stress, the active sites of PDI undergo an aberrant post-translational modification—S-nitrosylation—which prevents its normal enzymatic function (Forrester et al., 2006). Specifically, s-nitrosylation is the covalent addition of a nitric monoxide group to a cysteine thiol on PDI's active site, hence inhibiting its normal protective function, and resulting in the accumulation of misfolded proteins (Forrester et al., 2006). Uehara et al. (2006) demonstrated that PDI is s-nitrosylated in AD and PD patient brains, but not in that of healthy controls. Similarly, s-nitrosylated PDI levels in the lumbar spinal cords of ALS patients were approximately 5-fold greater compared to those of controls (Walker et al., 2010). S-nitrosylated PDI was also reported both in the brains of PrP^Sc^ infected rodents and in cell models of CJD bearing PrP^Sc^ misfolded proteins (Wang et al., 2012). Wu et al. (2014) investigated the effects of methamphetamine in cellular models of PD on the basis that methamphetamine users pose a higher risk of developing neurodegenerative disorders. A significant increase in the levels of nitric oxide synthase (NOS), NO and α-synuclein 24 h after methamphetamine treatment was reported in these cells (Wu et al., 2014). These changes to the nitrosative state of the cells resulted in the augmented aggregation of α-synuclein and the s-nitrosylation of PDI, suggesting that PDI could be a potential target to prevent methamphetamine-induced neurodegeneration (Wu et al., 2014). Furthermore, S-nitrosylated PDI was detected following ischemia/reperfusion injury and the levels increased with the formation of mutant SOD1 aggregates in primary astrocytes (Chen et al., 2012). Similarly, S-NO PDI correlates with synphilin misfolding in Parkinson disease (Forrester et al., 2006). S-nitrosylation is also involved in the re-distribution of PDI away from the ER by reticulons which maintain the curvature of the ER (Bernardoni et al., 2013). The deletion of reticulon 4A is protective in mouse models of ALS (Yang et al., 2009).

#### PDI family members

**Figure 5:**
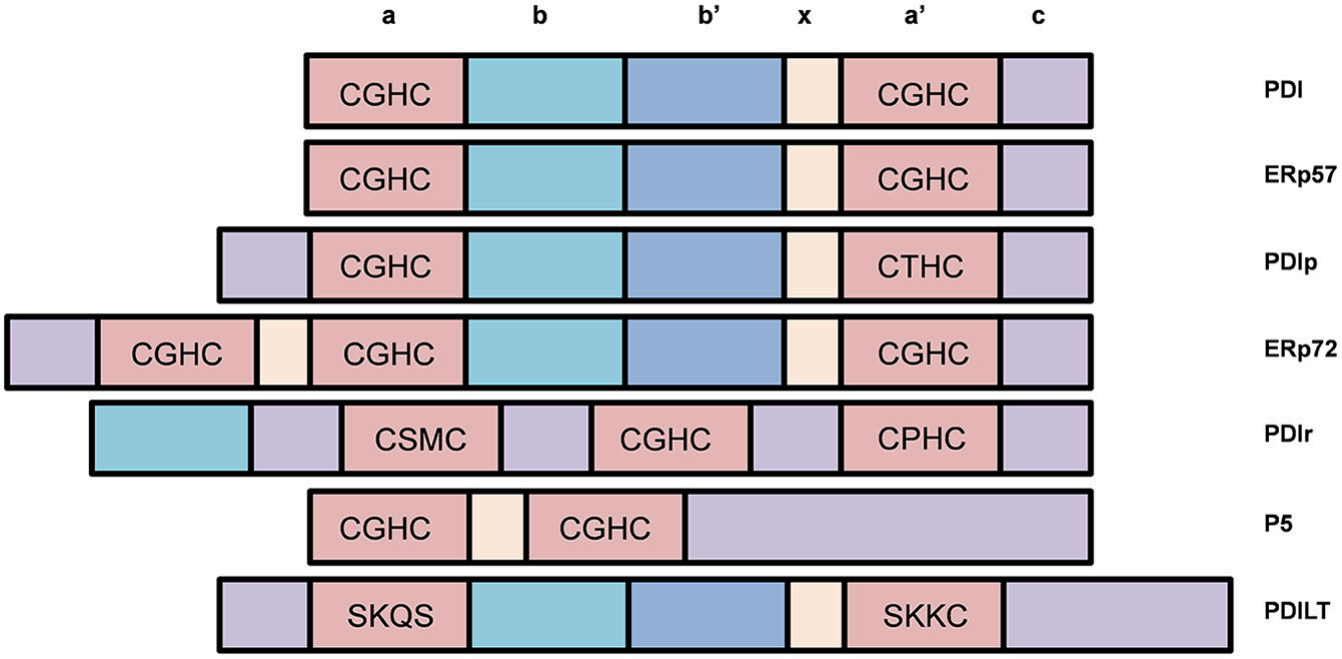
**Selected members of the Protein Disulfide Isomerase (PDI) family of chaperones and representation of their different domains**. Most PDI family members possess disulfide interchange activity and many contain the CGHC motif similar to that of PDI.

The PDI family contains over 21 structurally-related members that constitutes a network of proteins which promote the oxidative folding of multi-disulfide proteins (Kojima et al., 2014; selected members are highlighted in Figure 5). All members contain two or more thioredoxin-like active sites and the majority of members in this family also act as chaperones (Turano et al., 2002). The most common active site motif amongst PDI family members is CGHC, which is present in PDI, ERp57, and ERp72 (Figure 4). ERp57 is the closest known homolog of PDI. It has the same domain structure as PDI, but it lacks the C-terminal acidic region (Frickel et al., 2004).

## Evidence for induction of the UPR in neurodegenerative diseases

There is increasing evidence that activation of the UPR is a feature of most neurodegenerative disorders. The most obvious association between ER stress and neurodegeneration is via chronic UPR activation during disease, thus triggering apoptosis and neuronal cell death. The up-regulation of UPR markers in disease-affected neurons has now been described in cellular and animal models of disease, as well as in post-mortem human tissues, for most of these disorders. The initial participation of the UPR in pathogenesis might be neuroprotective as has been proposed in recent studies, but sustained activation of the UPR may subsequently initiate or accelerate neurodegeneration. In some instances the misfolded proteins are present within the cytoplasm rather than the ER, and can trigger ER stress by indirect mechanisms, that nevertheless disrupt ER homeostasis (Nishitoh et al., 2008; Atkin et al., 2014). Together, these studies suggest that the UPR pathway may be a potential therapeutic target for neurodegenerative diseases. Recent developments in the use of human neurons derived from reprogrammed induced pluripotent stem cells (iPSCs) provide a useful tool to unravel pathological mechanisms in these disorders, and ER stress and activation of the UPR are increasingly implicated in these studies (Table 2).

**Table 2:** **Evidence for ER stress and the role of PDI in neurodegenerative diseases**.

**Neurodegenerative disease**	**Protein inclusions**	**Evidence for ER stress**	**Evidence for a role of PDI**
Alzheimer's disease (AD)	β-Amyloid Tau	BiP, PERK, IRE1, and eIF2α upregulated in AD patients (Hoozemans et al., 2005, 2009). Cleaved caspases and JNK upregulated in AD patients (Ghribi et al., 2004; Lee et al., 2010a). CHOP upregulated in AD animal models and cell models treated with β-amyloid, and in AD patients (Ghribi et al., 2004; Chafekar et al., 2008; Lee et al., 2010a). IPSC-derived neurons and astrocytes from AD patients accumulate Aβ oligomers (Kondo et al., 2013).	PDI co-localizes with tau protein (Honjo et al., 2010). PDI levels increase 9.49 fold in tangle-bearing neurons in AD patients (Lee et al., 2010a). β-amyloid co-localizes with ERp57 (Erickson et al., 2005). Pharmacological activation of ERp57 reduces amyloid plaques and neurofibrillary tangles in brains, and improved object recognition memory in AD mouse models (Tohda et al., 2012).
Parkinson's disease (PD)	α-Synuclein	Upregulation of IRE1, PERK, eIF2α, and ATF4 in PD cell models (Ryu et al., 2002). Phosphorylated PERK and phosphorylated eIF2α detected in dopaminergic neurons of PD patients (Hoozemans et al., 2007). Co-localization of phosphorylated PERK and α-synuclein in dopaminergic neurons (Hoozemans et al., 2007). CHOP upregulation in dopaminergic neurons of mouse models and in cell models (Ryu et al., 2002; Holtz and O'Malley, 2003; Silva et al., 2005).	Upregulation of PDIA2 in PD cell models and post-mortem brain tissues of PD patients (Conn et al., 2004). PDIA2 immunoreactivity evident in Lewy Bodies (Conn et al., 2004). PDI upregulated in PD cell models (Ryu et al., 2002).
Amyotrophic lateral sclerosis (ALS)	SOD1 TDP-43 FUS C9orf72	UPR and CHOP induced prior to symptom onset in SOD1^G93A^ mouse models (Atkin et al., 2006; Kikuchi et al., 2006; Saxena et al., 2009). PERK, IRE1 and ATF6 upregulated in post-mortem human spinal cord tissues (Atkin et al., 2008). Deletion of BIM, XBP-1, ASK1, Puma or ATF4 delays disease onset or extends survival in ALS mouse models (Kieran et al., 2007; Nishitoh et al., 2008; Hetz et al., 2009; Matus et al., 2013a,b). Pharmacological inhibition of eIF2α dephosphorylation extends survival of ALS mouse models (Boyce et al., 2005; Saxena et al., 2009).	PDI upregulated in SOD1^G93A^ mouse models at presymptomatic, symptomatic and end stages of disease (Atkin et al., 2006, 2008). PDI co-localizes with inclusions in ALS mouse models, cell models and ALS patients (Atkin et al., 2006, 2008; Tsuda et al., 2008; Honjo et al., 2011; Farg et al., 2012; Walker et al., 2013). PDI and ERp57 upregulated in ALS patient and mouse model spinal cord tissues and patient CSF (Atkin et al., 2006, 2008). PDI over expression decreases mutant SOD1 inclusion formation, BiP and CHOP expression and PERK phosphorylation in ALS cell models (Walker et al., 2010). PDI knock down increases mutant SOD1 inclusion formation in ALS cell models (Walker et al., 2010).
Huntington's disease (HD)	Huntingtin	BiP and CHOP upregulated in post-mortem brains from HD patients and in HD cell models (Duennwald and Lindquist, 2008; Carnemolla et al., 2009). BIM upregulated in HD animal and cells models (García-Martínez et al., 2007; Kong et al., 2009; Leon et al., 2010). XBP-1 upregulated in striatum of HD patients (Vidal et al., 2012). XBP-1 knock down reduced neuron loss and mHtt levels, and improved motor performance in HD mouse models (Vidal et al., 2012).	Increased basal expression of PDI in HD cell models (Duennwald and Lindquist, 2008). PDI elevated in hippocampus of HD mouse models (Safren et al., 2014).
Creutzfeldt-Jakob disease (CJD)	Prion protein	Upregulation of caspase-12 in CJD cell models and post-mortem patient cortex tissues (Hetz et al., 2003). Upregulation of ERp57, Grp94 and BiP in HD cell models and patient cortex tissues (Hetz et al., 2003). Increased intracellular calcium release from the ER (Torres et al., 2010).	PDI overexpression in brains of CJD patients (Yoo et al., 2002). Increased expression of ERp57 (Hetz et al., 2005). ERp57 overexpression protects cells from PrP^sc^ toxicity and decreases rate of caspase-12 activation (Hetz et al., 2005).

### Alzheimer's disease

Whilst initial reports were contradictory, there is now convincing evidence for ER stress and UPR activation in AD, with recent studies suggesting that ER stress has a fundamental role in AD etiology. Hoozemans et al. (2005) demonstrated that the expression of ER chaperone, BiP, and the activated, phosphorylated form of UPR sensor PERK, were significantly increased in the temporal cortex and hippocampus in AD patients compared to controls. Furthermore, in a later study the same group reported upregulation of PERK, IRE1 and eIF2α in neurons of the hippocampus in AD patients; in particular, in neurons with granulovacuolar degeneration (Hoozemans et al., 2009). Evidence for pro-apoptotic UPR in AD was reported by Ghribi et al. (2004), when CHOP and JNK were up-regulated in animal disease models. Similarly, CHOP activation was detected in neuronal cells treated with Aβ (Chafekar et al., 2008). Lee and colleagues (2010a) detected increased levels of cleaved caspase-12, cleaved caspase-3, cleaved caspase-4, and CHOP in the temporal cortex of AD patients. *In vitro* studies involving neuronal cells in culture exposed to Aβ oligomers, suggested that the ensuing activation of ER stress correlates with the induction of Tau phosphorylation, thus providing an interesting link between ER stress, Aβ-mediated neurotoxicity and Tau hyperphosphorylation (Resende et al., 2008; Ferreiro and Pereira, 2012). However, whilst UPR activation can induce Tau phosphorylation, Tau phosphorylation may not activate ER stress (Sakagami et al., 2013). Modulation of the UPR can also be protective in AD cellular and animal models. PERK knockdown in neuronal cells enhanced Aβ toxicity through reduced activation of eIF2α (Lee et al., 2010b). Furthermore, activating the eIF2α pathway with Salubrinal significantly reduced caspase-4 dependent apoptosis in Aβ treated neurons (Lee et al., 2010b). These results suggest that the PERK-eIF2α pathway may play a role in cell survival, rather than apoptosis during ER stress. Interestingly, pathological events described in AD, such as neurofibrillary tangles, neuroinflammation, altered calcium signaling, and excitotoxicity, were also recently linked to the occurrence of pathological ER stress (Cornejo and Hetz, 2013). Similarly, IPSC-derived neurons and astrocytes from APP-linked familial and sporadic AD patients accumulated Aβ oligomers, leading to ER and oxidative stress (Kondo et al., 2013). ER stress was recently shown to promote cholesterol synthesis and mitochondrial cholesterol trafficking in AD mouse models (Barbero-Camps et al., 2014). It has also been proposed that ER stress may interfere with the normal trafficking of APP through the normal secretory pathway, leading to the production of Aβ, and subsequent toxicity (Plácido et al., 2014).

### Parkinson's disease

There is compelling evidence that ER stress is linked to death of dopamine neurons in PD, although most of this has been obtained from cell culture studies. However, in the substantia nigra of PD patients, Hoozemans et al. (2007) demonstrated immunoreactivity for PERK and eIF2α in their phosphorylated, active forms in dopaminergic neurons. Moreover, phosphorylated PERK was co-localized with increased α-synuclein immunoreactivity in dopaminergic neurons, suggesting a close association between UPR activation and the aggregation of α-synuclein (Hoozemans et al., 2007). Ryu et al. (2002) demonstrated that IRE1 and PERK were up-regulated in cell culture models that mimic the selective dopaminergic neuron degeneration that occurs in PD, as well as downstream targets, eIF2α, ATF4 and CHOP. Holtz and O'Malley (2003) screened dopaminergic neuroblastoma MN9D cells exposed to either 6-OHDA or MPP^+^ and noted that the most highly expressed transcript in both cases encodes for CHOP. Supporting this finding, Silva et al. (2005) demonstrated induction of CHOP in dopamine neurons of the substantia nigra in mouse models following intrastriatal injection of 6-OHDA (Silva et al., 2005). Similarly, overexpression of BiP or pharmacological modulation of the UPR in α-synuclein transgenic mice was protective (Colla et al., 2012; Gorbatyuk et al., 2012). More recently, cortical neurons generated from iPS cells of patients with α-synuclein mutations, identified ER stress as an early pathogenic phenotype (Chung et al., 2013). Interestingly, another recent study detected aberrant modification of ER stress sensors IRE1α and PERK by NO-mediated S-nitrosylation, in cell based models of PD. This resulted in loss of normal enzymatic function, leading to dysfunctional ER stress signaling and neuronal cell death Nakato et al. (2015). In contrast, other studies have demonstrated that some aspects of the UPR may be protective in PD. Overexpression of XBP-1 in MPP^+^-induced cell models was protective by suppressing apoptosis in cells exposed to proteasome inhibitors (Sado et al., 2009). Similarly, transplanting neural stem cells into the right lateral ventricles of rodents with rotenone-induced PD resulted in higher rates of survival in XBP-1 transfected neural stem cells compared to non-transfected cells (Si et al., 2012). Additionally, dopamine levels in the substantia nigra were significantly increased, α-synuclein expression was decreased, and neurological symptoms were significantly improved, following the transplantation of XBP-1 transfected neural stem cells (Si et al., 2012). The results of these studies suggest that XBP-1 enhancement is a possible therapeutic strategy for PD.

### Amyotrophic lateral sclerosis

Activation of the UPR is now well documented in cellular and animal models of ALS and in human patient tissues. Studies of transgenic mice expressing mutant SOD1^G93A^, a widely used disease model, revealed that UPR sensors, chaperones and apoptotic effectors were up-regulated in lumbar spinal cords during disease (Atkin et al., 2006; Kikuchi et al., 2006). Furthermore, the UPR was induced 60 days prior to symptom onset, and was present initially in those subtypes of motor neurons that degenerate first in ALS, indicating an active role for ER stress in pathogenesis (Saxena et al., 2009). Whilst SOD1 mutations represent only 2% of all ALS, and may not accurately represent pathology in the more common forms of disease, similar findings were obtained in post-mortem human spinal cord tissues of sporadic ALS patients (Ilieva et al., 2007; Atkin et al., 2008; Ito et al., 2009), thus placing ER stress on a more generic pathophysiology for ALS. For example, PERK, IRE1, and ATF6 are all upregulated in post-mortem human spinal cord tissues (Atkin et al., 2008). More recently, ER stress has been detected in neuronal cells expressing ALS-associated mutants of FUS and TDP-43 (Farg et al., 2012; Walker et al., 2013) and in animal models based on TDP-43 (Walker et al., 2013). Similarly, the UPR is induced in cell culture by ALS-associated mutant VAPB (Suzuki et al., 2009) and hexanucleotide repeat expansions in C9ORF72 (Zhang et al., 2014). Several mechanisms have been proposed for induction of ER stress in ALS, including impairment of ERAD by binding to Derlin-1 (Nishitoh et al., 2008) or impairment of protein transport between the ER and Golgi apparatus (Sundaramoorthy et al., 2013; Atkin et al., 2014). These studies implicate triggering of ER stress from the cytoplasm rather than the ER, although mutant TDP-43 and mutant FUS were recently shown to be associated with the ER (Soo et al., 2015) The same study also demonstrated that overexpression of Rab1, an intracellular vesicle trafficking regulator which plays a central role in UPR homeostasis, prevented ER stress in cells expressing mutant SOD1, TDP-43 and FUS. Furthermore, the presence of Rab1-positive inclusions in the motor neurons of human spinal cord tissues from sALS patients implies that Rab1 is misfolded and loses its normal vesicular distribution in sALS (Soo et al., 2015). Interestingly, Rab1 dysfunction has also been linked to PD (Cooper et al., 2006). In more recent studies, using reprogrammed IPS cells, ER stress was closely associated with the electrical excitability of motor neurons (Kiskinis et al., 2014), and that hyperexcitability may trigger ER stress (Wainger et al., 2014).

Several studies have demonstrated that modulation of the UPR genetically is protective in animal models of ALS. Deletion of BIM, XBP-1, ASK1, Puma or ATF4 either delays disease onset (Kieran et al., 2007; Matus et al., 2013a,b) or extends survival in transgenic mutant SOD1 mice (Nishitoh et al., 2008; Hetz et al., 2009). Similarly, pharmacological modulation of the UPR is protective in SOD1^G93A^ mice (Saxena et al., 2009), and either *C.elegans* and zebrafish expressing mutant TDP-43 (Vaccaro et al., 2013). However, SOD1^G85R^ mice with hemizygous deletion of PERK had a substantially accelerated disease onset and shortened lifespan compared to SOD1^G85R^/PERK^+∕+^ controls (Wang et al., 2011), indicating that some aspects of UPR induction are protective against disease. Similarly, pharmacological inhibition of eIF2α dephosphorylation using salubrinal delays disease and extends survival of SOD1^G93A^ mice (Boyce et al., 2005; Saxena et al., 2009) and decreased GADD34 slows disease progression and extends survival in this animal model (Wang et al., 2013). Together, these findings imply that PERK is a mediator of motor neuron survival in ALS, possibly by decreasing protein misfolding (Wang et al., 2011, 2013) or by inducing autophagy (Hetz et al., 2009). These results therefore highlight the opposing protective and pro-apoptotic properties of the UPR and suggest that selective targeting of specific components of the UPR could be beneficial in ALS.

### Huntington's disease

Evidence of induction of ER stress in human HD patients was provided by Carnemolla et al., where BiP and CHOP were up-regulated in post-mortem brains from HD patients (Carnemolla et al., 2009). Similarly, increased expression of XBP-1 was detected in the striatum of HD cases, although other markers (CHOP, ATF4, and GRP78) were not elevated (Vidal et al., 2012). ER stress is also detected early in HD mouse models and persists throughout the lifespan of these animals, similar to ALS rodent models (García-Martínez et al., 2007; Duennwald and Lindquist, 2008; Carnemolla et al., 2009). Duennwald and Lindquist (2008) demonstrated that in a striatal cell line derived from Htt knock-in mice, increased basal expression of UPR proteins BiP, CHOP and PDI was observed compared with control cells (Duennwald and Lindquist, 2008). The same study showed that toxic polyglutamine expansion repeats impaired ERAD and degradation pathways (Duennwald and Lindquist, 2008). This suggests that the polyglutamine repeat expansion of mutant Huntingtin compromises the proteasomal degradation of misfolded proteins in the ER, thus giving rise to ER stress. The induction of the pro-apoptotic protein BIM has also been linked to HD in both animal (García-Martínez et al., 2007) and cellular disease models (Kong et al., 2009; Leon et al., 2010). Additionally, caspase-12 and the JNK pathway were activated in cells expressing expanded polyglutamine aggregates. These data together suggest ER stress is linked to cell death in HD (Kouroku et al., 2002). ER stress has also been linked to motor phenotypes in HD. Silencing XBP-1 expression in mutant Htt (mHtt) transgenic mouse strain YAC128 reduced the loss of neurons in the striatum, decreased mHtt levels, and improved motor performance (Vidal et al., 2012). Conversely, ATF4 deficiency did not alter mHtt levels, highlighting the involvement of XBP-1 in HD pathogenesis (Vidal et al., 2012). Hence, whilst the XBP-1 pathway of the UPR is protective in PD, the opposite appears to be true in HD.

### Creutzfeldt-Jakob disease

Upregulation of caspase-12, ERp57, Grp94, and BiP was described by Hetz and colleagues in the cortex of post-mortem sporadic CJD and variant CJD patients compared to controls (Hetz et al., 2003). However, whether caspase-12 plays a role in neurodegeneration is controversial. Nevertheless, in neuronal cell cultures, PrP^Sc^ toxicity in CJD is associated with an increase in the release of intracellular calcium from the ER and the upregulation of ER chaperones, indicating a role for ER stress in prion diseases (Hetz et al., 2003; Torres et al., 2010). Similarly, prion replication and the expression of mutant PrP dysregulated ER calcium homeostasis, giving rise to ER stress in cell culture (Torres et al., 2010). Finally, another study showed that treatment of Neuro-2A cells with PrP^Sc^ resulted in the activation and upregulation of caspase-12 and significant upregulation of ER chaperones, ERp57, Grp94, and BiP (Hetz et al., 2003).

## PDI in neurodegenerative diseases

As PDI can facilitate protein folding, it is not surprising that PDI is increasingly implicated in neurodegenerative diseases where protein misfolding is a key component (summarized in Table 2). PDI is often found co-localized with misfolded proteins in disease-affected tissues, implying a possible role for PDI in preventing protein misfolding (Atkin et al., 2006; Honjo et al., 2010, 2011; Farg et al., 2012; Walker et al., 2013). In some diseases, there is direct evidence that PDI prevents aggregation and associated-toxicity, thus raising the likelihood that PDI is a possible therapeutic target in neurodegeneration. Interestingly, a recent study provided evidence that PDI family member Erp57 can also mediate neurite outgrowth in neurons, thus adding further complexity to the functions of PDI in relation to neurodegeneration (Castillo et al., 2015). However, PDI is often S-nitrosylated in these disorders, which would prevent its normally protective function (Uehara et al., 2006).

### PDI in Alzheimer's disease

Honjo et al. (2010) identified neurofibrillary tangles in the brains of patients with AD in which PDI was co-localized with tau. The levels of PDI were also markedly increased (up to 9.5 fold) in neurofibrillary tangle-bearing neurons in AD brains compared with those of age-matched controls and immunohistochemistry showed that PDI was primarily expressed in temporal cortex neurons in AD patients (Lee et al., 2010a). Immunoblotting studies of cerebrospinal fluid (CSF) from control patients indicated that a vast concentration of β-amyloid is normally bound to ERp57, forming a ERp57-β-amyloid complex (Erickson et al., 2005). This finding therefore implies that PDI family members normally prevent the aggregation of β-amyloid. Consistent with this notion, pharmacological activation of ERp57 using Disogenin significantly reduced amyloid plaques and neurofibrillary tangles in the cerebral cortex and hippocampus of a mouse model of AD, in which five familial AD human APP mutations were co-expressed (Tohda et al., 2012). Moreover, performance of object recognition memory was significantly improved in this mouse model, providing further evidence for a protective role for ERp57 (Tohda et al., 2012).

### PDI in Parkinson's disease

Conn et al. (2004) demonstrated an upregulation of PDI family member PDIA2 in SH-SY5Y human neuroblastoma cells exposed to MPP^+^, but not other family members PDI, ERp57 and ERp72. Similarly, PDIA2 was upregulated in post-mortem brain tissues from PD patients and immunohistochemical studies demonstrated that PDIA2 immunoreactivity was evident in Lewy bodies of these patients (Conn et al., 2004). Unlike in SH-SY5Y cells, PDI is upregulated in PC12 cells exposed to MPP^+^, rotenone and 6-Hydroxydopamine (Ryu et al., 2002).

### PDI in amyotrophic lateral sclerosis

PDI is upregulated in the spinal cords of SOD1^G93A^ mouse models of ALS at presymptomatic (p60), symptomatic (p90), and end stages (p120) of disease, and in human patient spinal cords (Atkin et al., 2006, 2008). Similarly, ERp57 is upregulated in SOD1^G93A^ mouse models at similar time points and in human patient tissues (Atkin et al., 2006). Furthermore, PDI associates with abnormal inclusions in SOD1^G93A^ mouse models and neuronal cells in culture (Atkin et al., 2006), as well as in ALS patients (Atkin et al., 2008). PDI also co-localizes with inclusions formed by other ALS-linked proteins, TDP-43 (Honjo et al., 2011; Walker et al., 2013), FUS (Farg et al., 2012) and vesicle associated membrane protein (VAPB) (Tsuda et al., 2008), implying that PDI is linked to general protein misfolding in ALS. PDI over-expression decreased mutant SOD1 aggregation and inclusion formation in neuronal cells and decreased BiP and CHOP expression as well as PERK phosphorylation, in comparison to controls, indicating that PDI is also protective against ER stress (Walker et al., 2010). Furthermore, knock down of PDI increased the formation of mutant SOD1 inclusions (Walker et al., 2010). These data together suggest a protective role for PDI against abnormal protein aggregation and ER stress in ALS. This neuroprotection is further supported by the deletion of a PDI regulator, Reticulon-4A, which accelerates the degeneration of motor neurons in SOD1 mice models (Yang et al., 2009). Quantitative western blotting also revealed an upregulation of PDI in the CSF of ALS patients in comparison to controls (Atkin et al., 2008). This finding may explain why PDI is subsequently found in numerous cellular locations and secreted by various cell types, instead of being localized exclusively to the ER (Turano et al., 2002).

The profile of PDI in ALS has increased recently by the identification of PDI variants as a genetic risk factor for the disease. Kwok et al. (2013) reported that single nucleotide polymorphisms (SNPs) in the P4HB gene encoding PDI were associated with fALS and sALS. They reported significant genotypic associations for two SNPS, rs876016, and rs2070872, in fALS and significant allelic associations for rs876016 with both sporadic and familial forms, suggesting that these SNPs are risk factors for ALS (Kwok et al., 2013). Additionally, a more recent study by Yang and Guo (2015) examined these same two SNPs in sALS patients in the Chinese Han population. They demonstrated a significant association of these SNPs with sALS, implying that genetic variants in the P4HB gene may be a contributing factor for sporadic forms of ALS in the Chinese Han population. A further study by Gonzalez-Perez et al. (2015) identified 16 variants in PDI and ERp57, with 1-2% present in all fALS and 1% present in all sALS cases analyzed. This frequency is similar to that of other ALS-linked gene variants (Turner et al., 2013). Structural analysis of PDI variants predicted a change in the catalytic functioning of these proteins, and changes in the structure of ERp57 variants are thought to affect the calnexin-calreticulin cycle (Gonzalez-Perez et al., 2015).

### PDI in other neurodegenerative diseases

Upregulation of PDI is also implicated in HD and prion encephalopathies. Cells expressing polyglutamine expansion Htt repeats exhibited elevated PDI levels when compared to control cells expressing Htt with 25 repeats (Duennwald and Lindquist, 2008). Similarly, PDI was elevated in the hippocampus of transgenic mouse models of HD when compared to wildtype mice (Safren et al., 2014).

Yoo et al. (2002) observed an overexpression of PDI in the brains of CJD patients. Hetz et al. (2005) observed an upregulation of ERp57 in PrP^sc^ toxicity and also reported that ERp57 overexpression protected cells against PrP^sc^ toxicity and decreased the rate of caspase-12 activation (Hetz et al., 2005). Similarly, inhibition of ERp57 expression led to a significant increase in PrP^sc^ toxicity (Hetz et al., 2005). A study by Wang et al. (2012) evaluated the levels of some PDI family members in brain tissues of rodents infected with scrapie strain 263K. Western blot analysis revealed a significant upregulation in the expression of PDI, ERp57 and BiP, and a significant decrease in the levels of caspase-3. Increases in PDI and BiP were also observed in cells expressing PrP mutants, and furthermore, overexpression of PDI reduced ER stress and cytotoxicity in these cell models (Wang et al., 2012).

### Recent developments in PDI function associated with neurodegeneration

Although PDI is generally associated with a protective effect in maintaining proteostasis, recent studies have suggested that in some circumstances, PDI activity can be detrimental and can even trigger apoptosis. This pro-apoptotic function of PDI is specifically associated with the presence of misfolded proteins: expression of mutant huntingtin resulted in accumulation of PDI at ER-mitochondrial junctions and apoptotic cell death (Hoffstrom et al., 2010). Furthermore, inhibitors specifically targeting PDI reduced cellular toxicity induced by mutant Huntington (Hoffstrom et al., 2010). These data therefore point to a novel mechanism linking protein misfolding to apoptotic cell death induced by PDI. Other recent studies suggest that PDI can induce oxidative stress. PDI interacts with NADPH oxidase and overexpression of PDI leads to increased levels of ROS and apoptosis (Paes et al., 2011). Over-expression of PDI also appears to induce oxidative stress in microglial cells in SOD1^G93A^ mice (Jaronen et al., 2013). The S-nitrosylation of PDI is also associated with potentially damaging consequences. Wang et al. (2012) found that S-NO PDI plays an essential role in the cytotoxicity induced by PDI. The opposing effects of PDI, both protective and harmful, were recently reviewed, and the reader is directed to this for further information (Parakh and Atkin, 2015).

## Conclusion

ER stress has been widely studied in neurodegenerative diseases, and emerging evidence highlights the complexity of the UPR in these disorders, with both protective and detrimental components being described. Despite having different clinical manifestations, neurodegenerative diseases are similar in pathology; that is, an accumulation of misfolded proteins in neurons and subsequent disruption to cellular proteostasis. The PDI proteins are a large family of chaperones with complex functions which offer the potential to be exploited therapeutically in the future. However, the great complexity of the ER within neurons, particularly in the dendrite and axonal compartments, is only just becoming realized. Further studies in this area are warranted before the true contribution of the UPR and ER homeostasis to pathology can be appreciated.

## Author contributions

EP wrote the manuscript with additional contributions and revisions from the other authors.

## Funding

This work was supported by funding from the National Health and Medical Research Council of Australia (Project grants 1006141, 1030513, and 1086887) and the Motor Neurone Disease Research Institute of Australia, Angie Cunningham Laugh to Cure MND Grant. EP is supported by an Australian Postgraduate Award scholarship.

### Conflict of interest statement

The authors declare that the research was conducted in the absence of any commercial or financial relationships that could be construed as a potential conflict of interest.
